# Combat-related bridge synostosis versus traditional transtibial amputation: comparison of military-specific outcomes

**DOI:** 10.1007/s11751-015-0240-4

**Published:** 2015-12-07

**Authors:** Benjamin F. Plucknette, Chad A. Krueger, Jessica C. Rivera, Joseph C. Wenke

**Affiliations:** MCHE-DOR-O Orthopaedic Surgery Residency, 3851 Roger Brooke Dr, JBSA-Ft. Sam Houston, TX 78234 USA; Extremity Trauma and Regenerative Medicine, USAISR, 3698 Chambers Pass, JBSA-Ft. Sam Houston, TX 78234 USA

**Keywords:** Transtibial, Combat related, Amputation, Ertl, Burgess, Outcomes, Military specific, Outcomes

## Abstract

The aim of our study was to determine military-specific outcomes for transtibial amputations of US Service members using either the traditional technique (Burgess) or the Ertl technique. All US Service members sustaining transtibial, combat-related amputation from September 2001 through July 2011 were reviewed. Amputation type, mechanism of injury, time interval to amputation, age, sex, branch of service, rank, force, nature, and injury severity score were recorded. Outcomes were determined by analyzing military-specific medical review results, to include the following: Physical Evaluation Board Liaison Office (PEBLO) rating (0–100), PEBLO outcome (permanent retirement, temporary disability retirement, separation without benefits, continuation of active duty, or fit for redeployment), and the rate of redeployment. Amputation type (Ertl vs. Burgess) was determined by reviewing postoperative radiographs and radiology reports. Data from all of the above categories were compared for both Ertl and Burgess amputees. Of 512 subjects identified, 478 had radiographs or radiology reports distinguishing between Ertl or Burgess transtibial amputation. A total of 406 subjects underwent the Burgess procedure, and 72 subjects underwent the Ertl procedure. There was not a significant difference between the two groups in review board rating (*p* = 0.858), review board outcome (*p* = 0.102), or ability to deploy (*p* = 0.106); however, subjects that underwent the Ertl procedure remained on active duty at a significantly higher rate (*p* = 0.021). There is a higher rate of remaining on active duty using the Ertl technique. This study suggests that there is an improvement in functional outcome with the Ertl technique.

**Electronic supplementary material:**

The online version of this article (doi:10.1007/s11751-015-0240-4) contains supplementary material, which is available to authorized users.

## Introduction

There is a high rate of lower extremity amputation within the combat-deployed population of the US military [1]. The Ertl synostosis technique [2] and the traditional Burgess technique [3] (where the tibia is cut and beveled—as is the fibula at a slightly more proximal level—leaving the proximal tibiofibula joint undisturbed) are two well-described forms of amputation. In comparison with the Burgess method, the Ertl technique utilizes either a section of fibula as a strut or a periosteal sleeve to bridge the distal aspect of the residual limb and create a platform synostosis on which to bear weight [4]. While there are proponents for each of the two amputation techniques, there is a paucity of outcomes-based research as to one technique being superior to the other.

Proponents of the Ertl technique claim that the bone bridge provides a more stable platform for prosthetic weight bearing [5]. Some studies claim there is greater residual limb health, improved prosthetic fit and a higher health-related quality of life in patients with an Ertl amputation [5, 6]. There are other studies refuting the Ertl amputation results in a superior bearing surface [7]. A fluoroscopic evaluation of prosthetic fit related to residual limb displacement also demonstrates no kinematic difference between the two amputation techniques, further disproving the theoretical benefit of the Ertl technique to improved fit of prosthesis [8]. Recent research on functional outcomes measures also shows no difference in military populations [9]. Critics of the Ertl amputation cite an increased operative time and complications as contraindications to creating a bone bridge in patients with an otherwise stable fibula [10, 11].

The aim of this study was to determine the characteristics and military-specific outcomes in US service members with either a Burgess or Ertl transtibial amputation, as determined by the military’s physical evaluation board disposition and the rate of deployment following amputation. The null hypothesis for this study was that there would be no difference in military-specific outcomes for patients undergoing the Ertl amputation versus the Burgess amputation.

## Materials and methods

A previously studied cohort of amputees was reviewed consisting of all US major extremity amputees (proximal to the metacarpals or metatarsals) involved in Operation Iraqi Freedom, Operation Enduring Freedom and Operation New Dawn from September 2001 through July 2011 [1, 12]. All subjects who sustained a transtibial amputation and had data available regarding their return to duty status were examined. Type of amputation, mechanism of injury, time interval to amputation, age, sex, branch of service, rank, force, nature, and injury severity score were recorded. Amputation type (Ertl vs. Burgess) was determined by reviewing postoperative radiographs and radiology reports.

Military rank is determined by merit, time in service and presence or absence of a college degree at the time of joining. Those with a bachelor’s degree or higher at the time of joining are commissioned officers. Those who join without a college degree are enlisted members. Of the enlisted, those who demonstrate merit and serve long enough are promoted to non-commissioned officer (NCO); they are the more senior enlisted members.

Additional outcome measures were based on the results of each service member’s Physical Evaluation Board Liaison Office (PEBLO) review. The PEBLO is a group of medical personnel responsible for determining whether or not an injured service member is able to continue serving on active duty status and how much disability a service member should receive if they are determined to be unfit for duty. Each case handled by the PEBLO is unique and, while there are guidelines followed during the evaluation of injured service members, individuals with similar injuries may have different rulings on their status and disability by the PEBLO based on a multitude of factors. In addition, each service branch has their own guidelines regarding return to duty and injury compensation that are factored into PEBLO decisions. Once a service member has reached a point of maximal medical benefit following an injury as determined by their treating physician on a case by case basis, those that are deemed capable of performing military-specific duties are placed back on active duty status. The treating physician is not always clearly identified but is usually defined as the physician of the specialty responsible for the subject’s primary limitation in returning to duty. Service members that warrant further evaluation prior to returning to active duty are reviewed by the PEBLO to determine whether a disability persists that would limit active duty status. The PEBLO categorizes the service member into one of five categories: fit for duty (FIT), eligible for continuation on active duty (COAD) in a limited capacity or under a new occupational role, temporarily disabled retired list (TDRL), permanently retired (PR) or separate with severance pay (SWSP). Correlating to the civilian sector, FIT corresponds to returning to the original occupation regardless of job requirements; COAD corresponds to returning to the workforce in a limited or different capacity; TDRL corresponds to inability to return to the workforce in any capacity owing to a disability that may be permanent but has not had sufficient time to stabilize to determine ultimate disposition; PR corresponds to the inability to rejoin the work force in any capacity and 100 % disability; SWSP corresponds to the inability to rejoin the workforce in any capacity and a disability rating <30 %. The PEBLO also assigns each service member a disability rating that reflects how much his or her persistent cumulative disability detracts from their ability to perform military tasks. The overall disability rating takes into account all of the separate persisting conditions that limit a service member’s ability to return to duty; some injured service members may have one “disabling condition,” while others may have several conditions contributing to their overall disability. The disability rating (expressed as a percentage) also determines eligibility for disability benefits after medical discharge from active duty. For reference, an isolated-below-the-knee amputation usually carries a disability rating of 40. The disability rating is used to determine the percentage of disability payments a member will be eligible for after separation from the military. As the disability rating increases, a higher percentage of the full disability payment is allotted. A disability rating above 75 % denotes full disability, making the service member eligible for the maximum allotted disability payment.

Information pertaining to return to duty status, disabling conditions, disability ratings per disabling condition, military occupation status (MOS) and final total disability rating for each amputee was gathered from their PEBLO. The frequency of unfitting conditions and the average percent disability for each disabling condition were calculated. Deployment data were obtained from the Extremity Trauma and Amputation Center of Excellence (Fort Sam Houston, TX). Outcomes were determined by analyzing military-specific medical review results, to include the following: PEBLO rating (0–100), PEBLO outcome (PR, TDRL, SWSP, COAD or FIT) and the rate of deployment after amputation. Data from all of the above categories were compared between subjects that underwent the Ertl versus the Burgess amputation.

We noted that, in many cases, the radiographs following amputation were not available, but the radiology report was. To help determine whether the radiology reports were a reliable method to determine amputation type, we reviewed the radiology reports for a random sample of 20 subjects that had viewable radiographs demonstrating a previous Ertl procedure and gathered key terms from the associated radiology report that indicated the patient had undergone this specific procedure. All 20 of these subjects’ radiology reports contained some variation of one of the following phrases related to their amputation site: transverse bone graft, osseous or fibular strut, and endobutton or screw creating fusion. These key terms were then sought in the radiology reports of a separate sample of 20 subjects that we felt had undergone the Ertl procedure based on their radiology report alone as they were without viewable radiographs. These same phrases appeared in all 20 subjects’ reports in the second sample. The phrases were again sought in a third and fourth sample of 20 patients that we felt had undergone the Burgess procedure based on a viewable radiographs or radiology reports alone, respectively. These phrases were not found in the reports of any subject in the latter two samples. Based on this analysis, we feel that we have reliably categorized subjects into the correct amputation group based on, in many cases, radiology reports alone.

Statistical analysis was performed using publicly available Internet software (GraphPad Software, Inc, San Diego, CA, and Quantpsy.org, Nashville, TN). Categorical data comparing the Ertl to the Burgess amputation were completed using the Fisher’s exact or Chi-squared tests. Ranks were combined into ranges and tested via the Cochran–Armitage trend test. Continuous variables were analyzed using the Student’s *t* test. The limit for statistical significance was set at a two-tailed *p* value of 0.05.

## Results

**Fig. 1:**
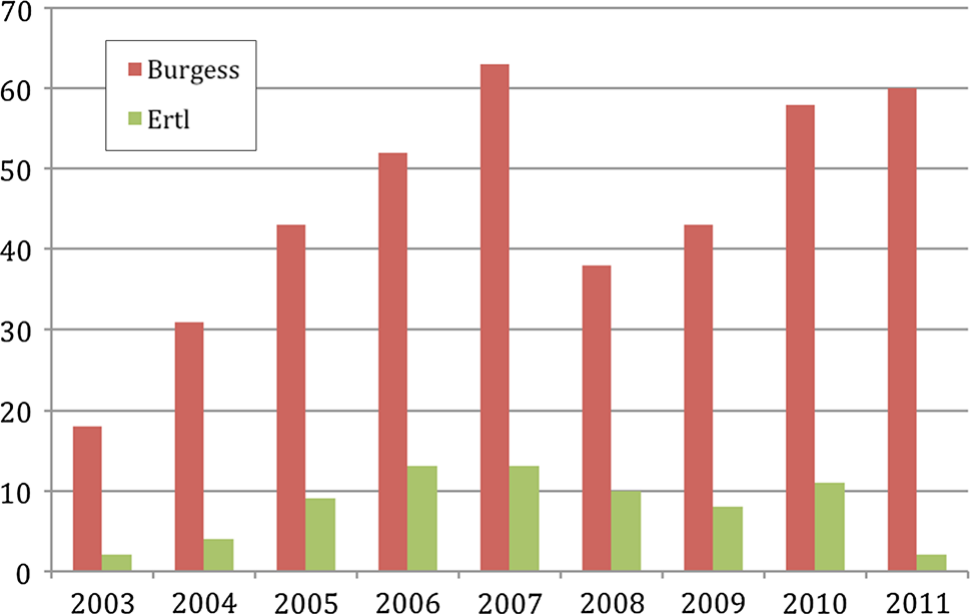
Frequency of amputation type by year

Of 512 subjects identified, 478 had radiographs or radiology reports distinguishing between Ertl and Burgess amputations. Thirty-four subjects were excluded for either lack of radiographs/reports or radiographs/reports that established that an Ertl or Burgess amputation was not present. Of the 478 subjects, amputation type was distinguished by radiographs in 155 and by radiology report in 323 (Table 1). Four hundred and six subjects underwent the Burgess amputation, and only 72 subjects underwent the Ertl procedure. There was no difference in the method of detection between groups (*p* = 0.924). Information regarding the frequency of each procedure per year is included in Fig. 1.

**Table 1:** Method of determining amputation type

Determination of AMP	Burgess	Ertl	Total
Radiology report	274 (67 %)	49 (68 %)	323 (68 %)
Radiograph	132 (33 %)	23 (32 %)	155 (32 %)
Total	406	72	478

Six of the total 478 subjects were female. Thirty-eight subjects were commissioned officers with the remaining subjects enlisted. There was a significant difference in the type of amputation based on rank with more officers undergoing the Ertl procedure (*p* = 0.019). The median age for both the Ertl and Burgess groups was 23 with ranges of 19–44 and 18–44, respectively. The population of this cohort matches that of many previous studies evaluating the combat-wounded; these were predominantly young, male, enlisted service members who served in the Army or Marines Corps as all but 20 of the amputations occurred in soldiers and marines, with airmen and sailors sustaining ten amputations per service. Complete demographic information is included in Table 2.

**Table 2:** Demographic information

	Burgess	Ertl	Total
Gender			
Female	5	1	6
Male	401	71	472
Total	406	72	478
Service			
Air Force	7	3	10
Army	284	57	341
Marine	106	11	117
Navy	9	1	10
Total	406	72	478
Age^a^			
Mean	25.16	25.21	
Median	23	23	
SD	5.36	5.07	
SEM	0.27	0.6	
N	406	72	
Rank^b^			
Jr Enlisted	128 (91 %)	13 (8 %)	141
NCO	248 (83 %)	51 (17 %)	299
Officer	30 (79 %)	8 (21 %)	38
Total	406	72	478

^a^ *p* = 0.941

^b^ *p* = 0.019

The majority of injuries were related to explosions (442/477) with the bulk of the remainder caused by gun shot wounds (23/477). The median ISS was 16.9 for the Burgess group and 14.6 for the Ertl group, which was not significantly different (*p* = 0.0598). Information regarding injury characteristics is displayed in Table 3.

**Table 3:** Injury characteristics

	Burgess	Ertl	Total
Class^a^			
Battle	395	70	465
Non-battle	11	2	13
Total	406	72	478
MOI^b^			
Bullet/GSW/firearm	17	6	23
Explosive device	379	63	442
Fall	2	2	4
Helo crash	3	0	3
MVC	5	0	5
Total	406	71	477
Force^c^			
Blunt	112	33	145
Burn	4	0	4
Penetrating	290	39	329
Total	406	72	478
ISS^d^			
Mean	16.9	14.61	
SD	9.36	10.1	
SEM	0.47	1.19	
N	404	72	

^a^Injury type classification for Burgess and Ertl subjects

^b^Mechanism of injury for Burgess and Ertl subjects

^c^Force of injury for Burgess and Ertl subjects

^d^Average injury severity score for Burgess and Ertl subjects, *p* = 0.060

Significantly more Burgess amputations were performed in the deployed setting (*p* = 0.002). The median time to amputation in the Ertl group was 258 days (range 0–2019 days) compared to 58 days (range 0–1281 days) in the Burgess group (*p* < 0.0001). Information regarding amputation characteristics is displayed in Table 4.

**Table 4:** Amputation characteristics

	Burgess	Ertl	Total
Days from injury to AMP^a^			
Mean	58.32	297.42	
SD	171.03	412.6	
SEM	8.49	48.63	
N	406	72	
AMP facility^b^			
War zone	161	15	176
Europe/USA	245	57	302
Total	406	72	478

^a^Average number of days from injury to amputation, *p* = 0.0001

^b^Location of amputation for Burgess and Ertl subjects, *p* = 0.002

There were no differences found regarding PEB rating (*p* = 0.858) or ability to deploy after amputation (*p* = 0.106). Time from injury to PEB date was longer in the Ertl group (*p* = 0.002). When considering every possible PEB outcome, there was no difference between groups (*p* = 0.102) but when grouping results based on ability to return to active duty (FIT + COAD), the Ertl group returned to active duty at as significantly higher rate (*p* = 0.021). The information regarding military-specific outcomes is displayed in Table 5.

**Table 5:** Military-specific outcomes

	Burgess	Ertl	Total
Days from amputation to PEB^a^			
Mean	595.59	771.96	
SD	356.25	404.75	
SEM	23.19	57.82	
N	236	49	
PEB rating^b^			
0	5	0	5
40	102	18	120
50	23	5	28
60	31	9	40
70	42	6	48
80	29	7	36
90	23	4	27
100	73	8	81
Total	328	57	385
PEB result^c^			
COAD	45	15	60
Fit	12	4	16
PR	219	33	252
SWSP	4	0	4
TDRL	39	8	47
Total	319	60	379
Return to duty^d^			
Yes	57	19	76
No	262	41	303
Total	319	60	379
Deployment^e^			
Yes	21	8	29
No	298	52	350
Total	319	60	379

^a^Average number of days from amputation to PEBLO evaluation 439, *p* = 0.002

^b^Comparison of PEBLO ratings for Burgess and Ertl subjects 440, *p* = 0.865

^c^PEBLO outcomes for Burgess and Ertl subjects 441, *p* = 0.102

^d^Ability to remain on active duty for Burgess and Ertl subjects 442, *p* = 0.021

^e^Ability to deploy after amputation for Burgess and Ertl subjects, *p* = 0.106

## Discussion

Amputation is one of the oldest forms of treatment for limb-threatening lower extremity injuries [13, 14]. There is a lack of consensus within the medical community regarding both the level and technique for amputation [13]. Despite the changes in technique and seemingly logical improvement in distribution of weight-bearing forces associated with the Ertl amputation, there remains a paucity of evidence to support the superiority of the Ertl technique versus the Burgess technique as to functional outcome. In a retrospective cohort study of 137 patients, Tintle et al. [14] demonstrated a significantly higher rate of non-infectious complications as well as a higher reoperation rate with Ertl amputations compared to Burgess amputations at an average of 2 years of follow-up. Despite this established difference in complication rates and repeat surgery, the data presented herein suggest that the difference may not extend to military-specific functional outcomes. Keeling et al. [9] evaluated patient-reported outcomes in 65 active duty military subjects having undergone either the Burgess or Ertl amputation with an average duration of follow-up of 32 ± 22.7 months. Some subjects that underwent the Ertl procedure reported improved prosthetic comfort and performance anecdotally, but these reports were not statistically linked to functional results. The study concluded that the two techniques offered similar outcomes; this is not supported by the data of this study with regard to military-specific outcomes. The research by Keeling et al. suggests that despite the increased complication and reoperation rate associated with the Ertl procedure reported by Tintle et al., subjects have similar results with either procedure. The results of our study allow a different conclusion to be drawn; subjects that undergo the Ertl procedure have a higher likelihood of returning to active duty, as 46 % of subjects that underwent the Ertl procedure returned to active duty in comparison with 22 % of Burgess subjects.

One of the most important findings of this study is that of 478 transtibial amputations analyzed in this cohort, only 15 % (72) underwent an Ertl amputation. For a subject that has garnered much attention over the past decade in the literature and meetings alike, it is interesting that there are so few Ertl amputations being performed. Such a low rate of Ertl amputation may indicate that many orthopedic surgeons do not see the value in performing this amputation or that a patient’s physiology is not conducive to the procedure.

Military-specific outcomes are of value because of the tremendous cost and residual disability following war injury experienced by otherwise healthy young service members [15]. Deployment presents substantial physical demands and the ability to deploy implies a high level of physical function [16]. In contrast to deployment following amputation, return to duty following amputation is often in a different role which may be less demanding. PEBLO ratings and disposition results were similar between the two amputation groups, but the Ertl group demonstrated a higher likelihood for returning to duty. For military populations, the ability to deploy correlates with the civilian metric of return to work for physically demanding occupations. In contrast, the ability to return to duty without deploying often represents the acceptance of a lesser role, which is difficult to translate to the civilian sector, as there are a range of occupations of differing demands offered in the military. Despite this difficult translation, it is clear that returning to duty represents the ability to return to the workforce. It is unclear as to why subjects in the Ertl group, while having similar deployment rates and PEB ratings, are more likely to remain on active duty. While these are obviously important measures of function and outcome, there are many variables that contribute to the ability to deploy after amputation and it is unlikely that amputation technique is solely accountable.

Despite the mostly similar functional outcomes between the two groups, there were differences between the groups in time from injury to amputation and time from injury to PEB hearing. These differences should be considered along with the finding that a significantly higher proportion of Burgess amputations occurred on the battlefield, while a higher proportion of Ertl amputations occurred at major military installations in Europe or America. Typically, military personnel present to Forward Operating Bases (with orthopedic surgeons—ranging in training from generalists to all fields of orthopedic fellowship—and limited surgical capabilities on hand) directly from the scene and within minutes of their injury. The higher rate of Burgess amputations on the battlefield reflects only the index amputation, which was likely accompanied by a series of irrigation and debridements prior to definitive closure in Europe or America. Whether these patients went on to Burgess amputations owing to the technique of the index procedure or from a lack of a salvageable strut is unclear. Amputations occurring in Europe or America inherently delay time from injury to amputation and time from injury to PEB hearing because of the necessary time included for patient stabilization and transport from the battlefield. However, this inherent delay does not fully explain the differences between groups. The Ertl procedure is more demanding technically, and battlefield surgeries generally strive to achieve stabilization more than definitive fixation. This concept may help to further explain the differences in surgical timing between the groups as it seems logical that the more technically demanding surgery would be delayed until the patient was both more physiologically stable and in the ideal operative environment. We hypothesize that Ertl amputees may have had less severe extremity injuries that, despite similar ISSs to the Burgess group, allowed a period of attempted limb salvage prior to undergoing their amputation or may have been seen by multiple surgeons and only agreed to undergo an amputation once a surgeon agreed to perform the Ertl procedure. It also seems likely that given that officers were more likely to undergo the Ertl procedure, these patients with higher education levels would be willing to explore every option prior to arriving at a final surgical procedure. These are factors that could have influenced the outcomes that are not accounted for in the study. Regardless, the difference in time between injury and amputation between both groups suggests that Ertl group may have been a different cohort of amputees with different characteristics than the Burgess group. It is reasonable that subjects who waited longer for their amputation would also have a longer overall wait from time of injury to PEB hearing. However, there is a cost associated with this waiting (longer period of immobilization, delay in rehab, etc.), and it remains unclear whether such a cost is worthwhile considering the lack of differences found between the two groups in our study and others. Furthermore, those amputees who were willing to wait for a provider to perform the Ertl procedure would be more likely to accept the processes that are required to remain on active duty within the military. Such a selection bias could provide an explanation for the differences found between Ertl and Burgess amputations in regard to active duty status. Some amputees may have heard other amputees or surgeons suggest that undergoing an Ertl amputation would increase their ability to perform higher-level activities after amputation, and if this were the case, this would also bias our results.

There have been passionate debates using anecdotal data to both support and discourage use of the Ertl procedure. One of the most important overall findings of this study may be that of 478 transtibial amputations analyzed in this cohort, only 15 % (72) underwent an Ertl amputation. For a subject that has garnered much attention over the past decade in the literature and meetings alike, it is interesting that there are so few Ertl amputations being performed. Such a low rate of Ertl amputation may indicate that many orthopedic surgeons do not see the value in performing this amputation or that a patient’s physiology is not conducive to the procedure.

There are limitations within the research presented. Unfortunately, 12 out of 72 of the subjects that underwent the Ertl procedure continued to await their PEBLO result, which introduces a degree of participation bias and limits the power of the data. In contrast to the validated outcome measures used by Keeling et al., our outcomes were based on the PEBLO scores and results which is an indirect measure of outcome. Despite these limitations, both groups were subjected to the same systematic scoring making their similarities and differences valid. Although direct visualization of postoperative radiographs was sought for all subjects, in many cases the only means of distinguishing the type of amputation was via a radiology report. Unfortunately, operative reports are frequently not generated, unavailable or difficult to obtain for amputations performed on the battlefield or in Europe. Owing to the lack of operative reports, it is not possible to comment on surgeon rationale for choosing one amputation technique over the other. To address this limitation, we performed the analysis described in the materials and methods section. Outcomes research is strengthened by complication rates and we do not include any information regarding complication rates between the groups. Additionally, subgroup analysis has inherent limitations and the conclusion that Ertl subjects have a higher return to duty rate was obtained in this fashion. Finally, despite statistically similar ISSs between the groups, we did not analyze associated injuries which could have revealed a difference between the two cohorts to help explain the results.

## Conclusion

This study found that only 15 % of all combat-related transtibial amputations performed used the Ertl technique. While it is unknown why so few of the amputations performed on this cohort used the Ertl technique, it may call into question the significance of the debate between proponents of the Ertl and non-Ertl transtibial amputations. Subjects that underwent the Ertl procedure were more likely to continue active duty military service. This study suggests that there is an improvement in military-specific outcomes with the Ertl technique, but such findings are not definitive based on the retrospective nature of this study and theoretical differences between the cohorts.

## Electronic supplementary material

Below is the link to the electronic supplementary material.

Supplementary material 1 (PDF 281 kb)

Supplementary material 2 (RTF 6 kb)
