# Supplementary material for: Combat-related bridge synostosis versus traditional transtibial amputation: comparison of military-specific outcomes
Source: Strategies Trauma Limb Reconstr. 2015 Dec 7;11(1):5–11. doi: 10.1007/s11751-015-0240-4 (PMC4814387; doi:10.1007/s11751-015-0240-4)
Supplement: Supplementary file 1 — Supplementary material 1 (PDF 281 kb) [file 11751_2015_240_MOESM1_ESM.pdf]

Annu. Rev. Public Health 2002. 23:151–69
DOI: 10.1146/annurev.publheath.23.100901.140546
Copyright © 2002 by Annual Reviews. All rights reserved

THE IMPORTANCE OF THE NORMALITY ASSUMPTION IN LARGE PUBLIC HEALTH DATA SETS

Annu. Rev. Public. Health. 2002.23:151-169. Downloaded from www.annualreviews.org
by U.S. Army Medical Research & Material Command on 01/03/14. For personal use only.

Thomas Lumley, Paula Diehr, Scott Emerson, and Lu Chen
Department of Biostatistics, University of Washington, Box 357232, Seattle, Washington 98195; e-mail: tlumley@u.washington.edu

Key Words parametric, nonparametric, Wilcoxon test, rank test, heteroscedasticity

■ **Abstract** It is widely but incorrectly believed that the t-test and linear regression are valid only for Normally distributed outcomes. The t-test and linear regression compare the mean of an outcome variable for different subjects. While these are valid even in very small samples if the outcome variable is Normally distributed, their major usefulness comes from the fact that in large samples they are valid for any distribution. We demonstrate this validity by simulation in extremely non-Normal data. We discuss situations in which in other methods such as the Wilcoxon rank sum test and ordinal logistic regression (proportional odds model) have been recommended, and conclude that the t-test and linear regression often provide a convenient and practical alternative. The major limitation on the t-test and linear regression for inference about associations is not a distributional one, but whether detecting and estimating a difference in the mean of the outcome answers the scientific question at hand.

INTRODUCTION

It is widely but incorrectly believed that the t-test and linear regression are valid only for Normally distributed outcomes. This belief leads to the use of rank tests for which confidence intervals are very hard to obtain and interpret and to cumbersome data-dependent procedures where different transformations are examined until a distributional test fails to reject Normality. In this paper we re-emphasize the uncontroversial statistical facts that the validity of the t-test and linear regression in sufficiently large samples depends only on assumptions about the variance of the response and that violations of those assumptions can be handled easily for the t-test (and with slightly more difficulty for linear regression). In addition to reviewing the literature on the assumptions of the t-test, we demonstrate that the necessary sample size is relatively modest by the standards of today's public health research. This is true even in one of the most extreme kinds of data we have encountered, annualized medical costs. We should note that our discussion is entirely restricted

Annu. Rev. Public. Health. 2002.23:151-169. Downloaded from www.annualreviews.org
by U.S. Army Medical Research & Material Command on 01/03/14. For personal use only.

to inference about associations between variables. When linear regression is used to predict outcomes for individuals, knowing the distribution of the outcome variable is critical to computing valid prediction intervals.

The reason for the widespread belief in a Normality assumption is easy to see. If outcomes are indeed Normally distributed then several different mathematical criteria identify the t-test and ordinary least squares regression as optimal analyses. This relatively unusual convergence of criteria makes the Normal theory an excellent example in mathematical statistics, and leads to its popularity in both theoretical and applied textbooks. The fact that the Normality assumption is sufficient but not necessary for the validity of the t-test and least squares regression is often ignored. This is relatively unimportant in theoretical texts, but seriously misleading in applied books.

In small samples most statistical methods do require distributional assumptions, and the case for distribution-free rank-based tests is relatively strong. However, in the large data sets typical in public health research, most statistical methods rely on the Central Limit Theorem, which states that the average of a large number of independent random variables is approximately Normally distributed around the true population mean. It is this Normal distribution of an average that underlies the validity of the t-test and linear regression, but also of logistic regression and of most software for the Wilcoxon and other rank tests.

In situations where estimation and comparison of means with the t-test and linear regression is difficult because of extreme data distributions, it is important to consider whether the mean is the primary target of estimation or whether some other summary measure would be just as appropriate. Other tests and estimation methods may give narrower confidence intervals and more powerful tests when data are very non-Normal but at the expense of using some other summary measure than the mean.

In this review we begin by giving the statistical background for the t-test and linear regression and then review what the research literature and textbooks say about these methods. We then present simulations based on sampling from a large data set of medical cost data. These simulations show that linear regression and the t-test can perform well in moderately large samples even from very non-Normal data. Finally, we discuss some alternatives to the t-test and least squares regression and present criteria for deciding which summary measure to estimate and what statistical technique to use.

DEFINITIONS AND THEORETICAL ISSUES

Least-Squares Techniques

We will discuss first the two-sample t-test, and then linear regression. While the t-test can be seen as merely a special case of linear regression, it is useful to consider it separately. Some more details of the calculations and a review of the Central Limit Theorem can be found in Appendix 1.

Annu. Rev. Public. Health. 2002.23:151-169. Downloaded from www.annualreviews.org
by U.S. Army Medical Research & Material Command on 01/03/14. For personal use only.

The t-Test

Two different versions of the two-sample t-test are usually taught and are available in most statistical packages. The differences are that one assumes the two groups have the same variance, whereas the other does not. The t-statistic, which does not assume equal variances, is the statistic in Equation 1. In Appendix 1 we show that, because of the Central Limit Theorem, this is normally distributed with unit variance when the sample size is large, no matter what distribution Y has. Thus, this version of the t-test will always be appropriate for large enough samples. Its distribution in small samples is not exactly a t distribution even if the outcomes are Normal. Approximate degrees of freedom for which the statistic has nearly a t distribution in small samples are computed by many statistical packages.

$$t = \frac{\overline{Y}_1 - \overline{Y}_2}{\sqrt{\frac{s_1^2}{n_1} + \frac{s_2^2}{n_2}}}. \qquad 1.$$

We next mention the version of the t-statistic that assumes the variances in the two groups are equal. This, the original version of the test, is often used in introductory statistics because when the data do have a Normal distribution, the statistic in Equation 2 has exactly a t distribution with a known number of degrees of freedom. One would rarely prefer this statistic in large samples, since Equation 1 is more general and most statistical programs compute both versions. However, Equation 2 is useful in illustrating the problem of *heteroscedasticity*.

$$t = \frac{\overline{Y}_1 - \overline{Y}_2}{\sqrt{\frac{(n_1-1)s_1^2 + (n_2-1)s_2^2}{n_1+n_2-2}}\sqrt{\frac{1}{n_1} + \frac{1}{n_2}}}. \qquad 2.$$

Equation 2 differs from Equation 1 in combining the two group variances to estimate a pooled standard deviation. It is identical to that in Equation 1 if either $n_1 = n_2$ or $s_1^2 = s_2^2$. The two forms will be similar if n_1 and n_2 or σ_1^2 and σ_2^2 are similar, as is often the case. However, it is possible for them to differ in extreme situations. Suppose n_1 is much larger than n_2. In that case, the denominator of the t-statistic in Equation 1 can be seen to be primarily a function of s_2^2, while the denominator of the t-statistic in Equation 2 is primarily a function of s_1^2. If the variances in the two groups are different, this can result in the two t-statistics having different denominators. For example, if n_1 is ten times as big as n_2, and the two variances also differ by a factor of 10, then Equation 1 will still be appropriate but Equation 2 will be too small or too large by a factor of about 2, depending on which group has the larger variance. In such an extreme case, it would be possible to make an incorrect inference based on Equation 2. That is, the Central Limit Theorem guarantees that the t-statistic in Equation 2 will be normally distributed, but it may not have variance equal to 1. This is not a problem in practice because we can always use Equation 1, but severe heteroscedasticity will cause problems for linear regression, as is discussed below.

Annu. Rev. Public. Health. 2002.23:151-169. Downloaded from www.annualreviews.org
by U.S. Army Medical Research & Material Command on 01/03/14. For personal use only.

Linear Regression

As with the t-test, least-squares linear regression is usually introduced by assuming that Y is Normally distributed, conditional on X. This is not quite the same as saying that Y must be Normal; for example, Y for men and women could each have a different Normal distribution that might appear bimodal when men and women are considered together. That they were Normally distributed when controlling for sex would satisfy the usual Normality assumption. Normality is not required to fit a linear regression; but Normality of the coefficient estimates $\hat{\beta}$ is needed to compute confidence intervals and perform tests. As $\hat{\beta}$ is a weighted sum of Y (see Appendix 1), the Central Limit Theorem guarantees that it will be normally distributed if the sample size is large enough, and so tests and confidence intervals can be based on the associated t-statistic.

A more important assumption is that the variance of Y is constant. As with the t-test, differences in the variance of Y for different values of X (heteroscedasticity) result in coefficient estimates $\hat{\beta}$ that still have a Normal distribution; as with Equation 2 above, the variance estimates may be incorrect. Specifically, if the predictor X has a skewed distribution and Y has different variance for large and small values of X, the variance of $\hat{\beta}$ can be estimated incorrectly. This can be related to the conditions for t-test (2) to be incorrect by writing the t-test as a linear regression with a single binary predictor variable. A binary predictor X is skewed when the proportions p with $X = 0$ and the proportion $q = 1 - p$ with $\mathrm{X} = 1$ are different [the skewness is equal to $(q - p)pq$]. Thus the condition that X is skewed and Y is heteroscedastic in this linear regression is the same as the condition that n and σ^2 both differ between groups for the t-test. Modifications analogous to t-test$\{1\}$ to provide reliable inference in the presence of substantial heteroscedasticity exist but are not widely implemented in statistical software. In the case of the t-test, we saw that heteroscedasticity must be extreme to cause large biases; in our simulations below we examine this question further for linear regression.

LITERATURE REVIEW

An unwritten assumption of much of the literature on the t-test is that all two-sample tests are effectively testing the same null hypothesis, so that it is meaningful to compare the Type I and Type II error rates of different tests. This assumption is frequently untrue, and testing for a difference in means between two samples may have different implications than testing for a difference in medians or in the proportion above a threshold. We defer until later a discussion of these other important criteria for selecting an estimator or test. Most of the literature on the assumptions of the t-test is concerned with the behavior of the t-test in relatively small samples, where it is not clear if the Central Limit Theorem applies.

For linear regression, the statistical literature largely recognizes that heteroscedasticity may affect the validity of the method and non-Normality does not. The literature has thus largely been concerned with how to model heteroscedasticity

Annu. Rev. Public. Health. 2002.23:151-169. Downloaded from www.annualreviews.org
by U.S. Army Medical Research & Material Command on 01/03/14. For personal use only.

and with methods that may be more powerful than linear regression for non-Normal data. These issues are outside the scope of our review.

A number of authors have examined the level and power of the t-test in fairly small samples, without comparisons to alternative tests. Barrett & Goldsmith (4) examined the coverage of the t-test in three small data sets, and found good coverage for sample sizes of 40 or more. Ratcliffe (22) looked at the effect on the t distribution of non-Normality, and provided an estimate of how large n must be for the t-test to be appropriate. He examined sample sizes of up to 80 and concluded that "extreme non-Normality can as much as double the value of t at the 2.5% (one tail) probability level for small samples, but increasing the sample sizes to 80, 50, 30, and 15 will for practical purposes remove the effect of extreme skewness, moderate skewness, extreme flatness, and moderate flatness, respectively." We note that the one-tailed tests he studied are more sensitive to skewness than two-tailed tests, where errors in the two tails tend to compensate. Sullivan & d'Agostino (32) found that t-tests produced appropriate significance levels even in the presence of small samples (50 or less) and distributions in which as many as 50% of the subjects attained scores of zero.

Sawilowsky & Blair (23) examined the robustness of the t-test to departures from Normality using Monte Carlo methods in 8 data sets with sample sizes up to 120. They found the t-test was robust to Type II error. Sawilowsky & Hillman (24) showed that power calculations based on the t-test were appropriate, even when the data were decidedly non-Normal. They examined sample sizes up to 80.

The bootstrap (12) provides another method of computing confidence intervals and significance levels using the t-statistic. The bootstrap is a general-purpose method for estimating the sampling distribution of any statistic computed from independent observations. The sampling distribution is, by definition, the distribution of the statistic across repeated samples from the same population. The bootstrap approximates this by assuming that the observed sample is representative of the population and by taking repeated samples (with replacement) from the observed sample. The bootstrap approach usually requires some programming even in statistical packages with built-in bootstrap facilities [e.g., Stata (29) and S-PLUS (17)]. There is a wide theoretical and applied literature discussing and extending the bootstrap, much of which is summarized in books by Efron & Tibshirani (12) and Davison & Hinkley (9).

Bootstrapping for comparing means of non-Normal data has been evaluated in the context of cost and cost-effectiveness studies. Barber & Thompson (3) recommended a bootstrap approach for testing for differences in mean costs. They presented two examples, with sample sizes of 184 and 32 patients, respectively. In both cases, the p-values and the confidence intervals were very similar using the t-test and using the bootstrap procedure. Rascati et al. (21) concluded that the bootstrap was more appropriate, but they only examined the distribution of the cost data, not the more relevant sampling distribution of the mean.

In a practical setting, the t-test should be discarded only if a replacement can perform better, so comparisons with other tests are particularly important. Cohen &

Annu. Rev. Public. Health. 2002.23:151-169. Downloaded from www.annualreviews.org
by U.S. Army Medical Research & Material Command on 01/03/14. For personal use only.

Arthur (8) looked at samples of 25 per group and found that t-tests on raw, log, and square transformed data; the Wilcoxon test; and a randomization test all exhibited satisfactory levels of alpha error, with the randomization test and the t-test having the greatest power. Stonehouse & Forrester (30) found that the unequal-variance form of the t-test performed well in samples drawn from non-Normal distributions but with different variances and sample sizes. The Wilcoxon test did not perform as well. Zimmerman (34) compared the t-test to the Wilcoxon test when data were non-Normal and heteroscedastic and found that the t-test performed better than the Wilcoxon. Zimmerman & Zumbo (35) found that rank methods are as influenced by unequal variances as are parametric tests, and recommended the t-test. Skovlund & Fenstad (27) also found that the t-test was superior to the Wilcoxon when variances were different.

Theoretical results on the properties of the t-test are mostly over 30 years old. These papers mostly examine how the skewness and kurtosis of the outcome distribution affects the t-statistic in fairly small samples. In principle, they could be used to create a modified t-statistic that incorporated estimates of skewness and kurtosis. At least one such test (7) has achieved some limited applied use. The original references appear to be to Gayen (14) and Geary (15), who approximated the distribution of the t-statistic in non-Normal distributions. They were followed by other authors in producing better approximations for very small samples or extreme non-Normality.

In contrast to the t-test, there has been little empirical research into the behavior of linear regression for non-Normal data. Such research typically focuses on the effects of extreme outliers, under the assumption that such outliers are caused by errors or at least may be excluded from the analysis. When residuals are not Normally distributed, these robust regression methods may be useful for finding the line that best fits the majority of the data, ignoring some points that do not fit well. Robust regression methods do not model the mean of Y but some other summary of Y that varies from method to method. There is little literature on robust regression at an elementary level, but chapters by Berk (5) and Goodall (16) are at least addressed to the practising statistician rather than the theoretician.

Textbooks of biostatistics frequently describe linear regression solely in the context of Normally distributed residuals [e.g., Altman (2), Fisher & van Belle (13), Kleinbaum et al. (18)] where it is the optimal method for finding the best-fitting line; however, the least-squares method was invented as a nonparametric approach. One of the inventors, Legendre [quoted by Smith (28)], wrote,

> Of all the principles which can be proposed for that purpose, I think there is none more general, more exact, and more easy of application, that of which we made use in the preceding researches, and which consists of rendering the sum of squares of the errors a minimum.

Discussions of linear regression that do not suppose Normality are relatively rare. One from an impeccable statistical authority is that of Stuart et al. (31). More commonly, a Normality assumption is presented but is described as less important

than other assumptions of the model. For example, Kleinbaum et al. (18, p. 117) wrote,

> [Normality] is not necessary for the least-squares fitting of the regression model but it is required in general for inference making ... only extreme departures of the distribution of Y from normality yield spurious results.

This is consistent with the fact that the Central Limit Theorem is more sensitive to extreme distributions in small samples, as most textbook analyses are of relatively small sets of data.

Annu. Rev. Public. Health. 2002.23:151-169. Downloaded from www.annualreviews.org
by U.S. Army Medical Research & Material Command on 01/03/14. For personal use only.

SIMULATIONS

The simulations in much of the statistical literature we reviewed refer to sample sizes far smaller than those commonly encountered in public health research. In an effort to fill part of this gap, this section describes some simulations that we performed with larger samples. We used data from the evaluation of Washington State's Basic Health Plan, which provided subsidized health insurance for low-income residents, starting in 1989 (10, 19). The 6918 subjects in the study were enrolled in four health plans, 26% in a health maintenance organization (HMO) and 74% in one of three independent practice associations (IPA). Subjects were aged 0 to 65 (mean 23 years) and were followed for an average of 22 months (range 1 to 44 months). Length of follow-up depended on when the person joined the program relative to the end of the evaluation period, and is probably not related to the person's health. During the study period 79% used some services. As examples we use the variables "cost of outpatient care," age, sex, and self-rated general health. The last variable is abbreviated EVGFP, for "excellent/very good/good/ fair/poor."

Example of Central Limit Theorem

The Central Limit Theorem depends on the sample size being "large enough," but provides little guidance on how large a sample might be necessary. We explored this question using the cost variable in the Washington Basic Health Plan data. Annualized outpatient cost has a very long right tail, as shown in Figure 1. We truncated the histogram at $3000 so that the distribution for lower values could be seen, but use the full distribution in the following analysis. The actual costs ranged from $0 to $22, 452, with a mean of $389. The standard deviation is $895, standardized skewness is 8.8, and standardized kurtosis is 131.

Figure 2 shows the sampling distribution of 1000 means of random samples of size 65, 129, 324, and 487 from this very non-Normal distribution (approximately 1%, 2%, 5%, and 7.5% of the population). The graph shows a histogram and a smooth estimate of the distribution for each sample size. It is clear that the means are close to Normally distributed even with these very extreme data and with sample sizes as low as 65.

Annu. Rev. Public. Health. 2002.23:151-169. Downloaded from www.annualreviews.org
by U.S. Army Medical Research & Material Command on 01/03/14. For personal use only.

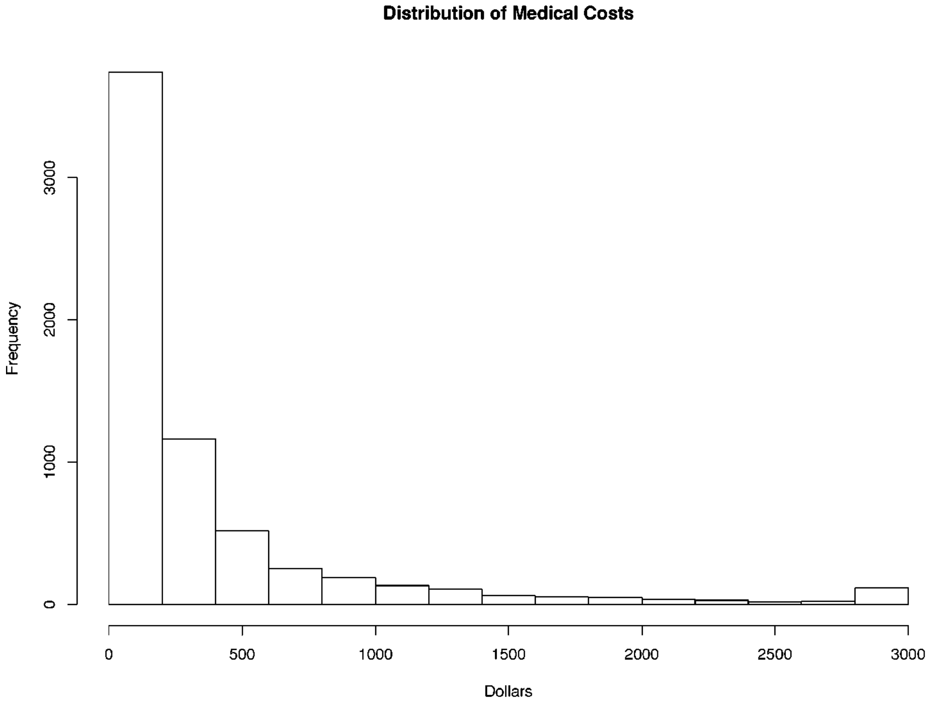

Figure 1 Distribution of annualized medical costs in the Washington Basic Health Plan.

Example for Linear Regression

Medical costs usually have the very non-Normal distribution we see here, but transformations are undesirable as our interest is in total (or mean) dollar costs rather than, say, log dollars (11). We considered the 6918 subjects to be the population of interest and drew samples of various sizes to determine whether the test statistics of interest had the distribution that was expected.

In addition, there is substantial heteroscedasticity and a somewhat linear relation between the mean and variance. In Figure 3 we divided subjects into groups by age and sex and calculated the mean and standard deviation of cost for each group. It is clear that the standard deviation increases strongly as the mean increases. The data are as far from being Normal and homoscedastic as can be found in any real examples.

We used these data to determine how large a sample would be needed for the Central Limit Theorem to provide reliable results. For example, as illustrated on the first line of Table 1, we drew 1000 1% samples, of average size 65, from the population. For each sample we calculated the regression of cost on age, sex, self-rated health, and HMO (IPA = 0) versus Fee for Service (IPA = 1). For each parameter in the regression model we calculated a 95% confidence interval and then checked

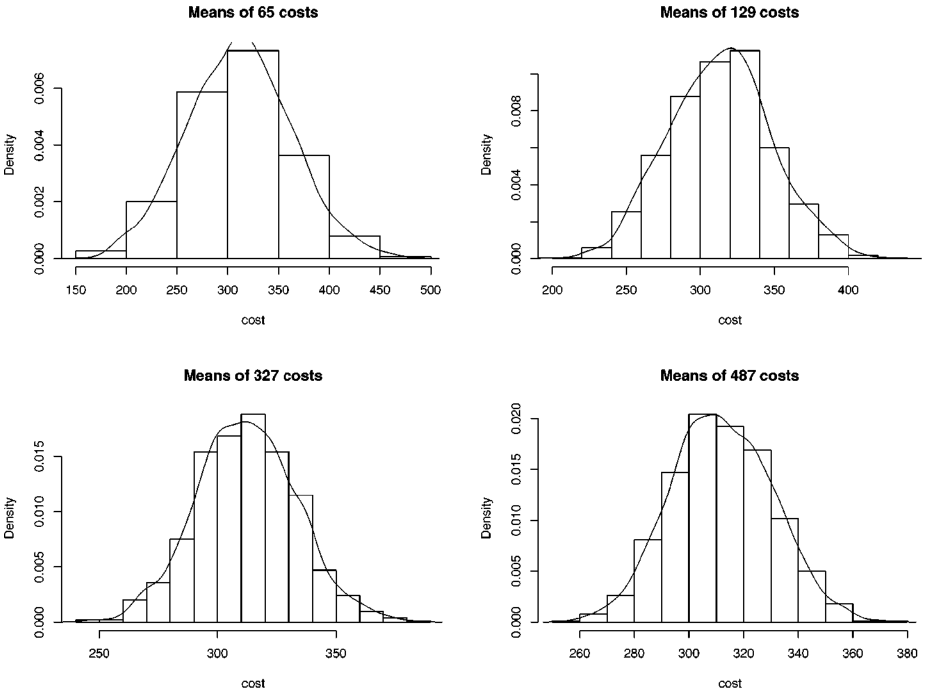

Figure 2 Distribution of means of samples of annualized costs.

Annu. Rev. Public. Health. 2002.23:151-169. Downloaded from www.annualreviews.org
by U.S. Army Medical Research & Material Command on 01/03/14. For personal use only.

to see whether the confidence interval contained the true value. The percent of times that the confidence interval included the value computed from the entire population of 6918 is an estimate of the true amount of confidence (coverage) and would be 95% if the data had been Normal to start with. For samples of size 65 and 129, some of the confidence interval coverages are below 90%. That means that the true alpha level would be 10% or more, when the investigator believed it to be 5%, yielding too many significant regression coefficients. Note that for sample sizes of about 500 or more, the coverage for all regression coefficients is quite close to 95%. Thus, even with these very extreme data, least-squares regression performed well with 500 or more observations.

These results suggest that cost data can be analyzed using least-squares approaches with samples of 500 or more. Fortunately, such large samples are usually the case in cost studies. With smaller samples, results for variables that are highly significant ($p < .001$, for example) are probably reliable. Regression coefficients with p-values between .001 and .10, say, might require additional analysis if they are important.

For data without such long tails much smaller sample sizes suffice, as the examples in the literature review indicate. For example, at one time a popular method of generating Normally distributed data on a computer was to use the sum

Annu. Rev. Public. Health. 2002.23:151-169. Downloaded from www.annualreviews.org
by U.S. Army Medical Research & Material Command on 01/03/14. For personal use only.

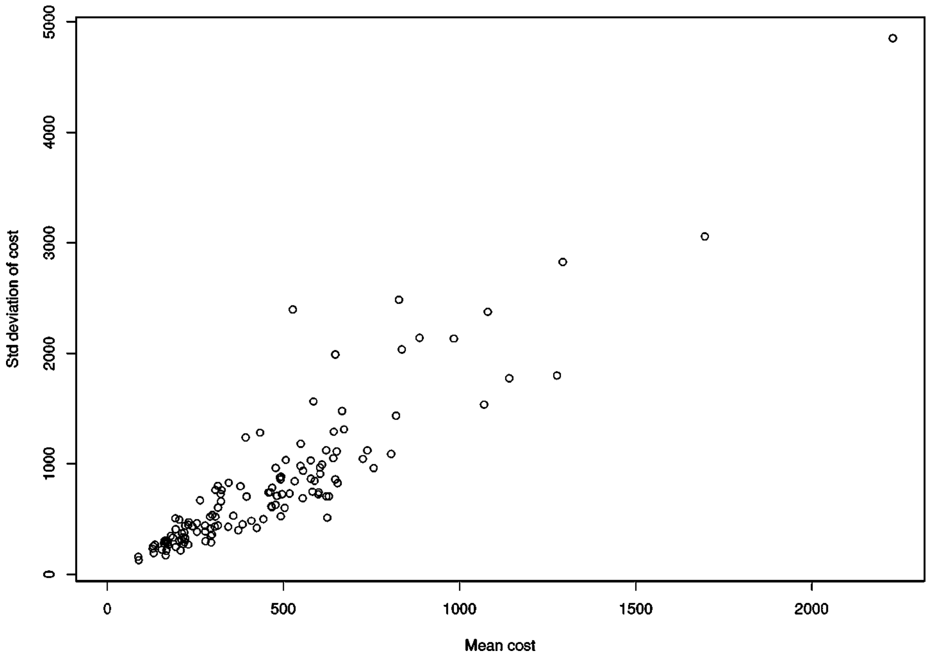

Figure 3 The relationship between mean and standard deviation of annualized costs, in age-sex subgroups.

of a sample of 12 uniformly distributed random numbers. The resulting distribution was not just close enough to Normal for statistical purposes, it was effectively indistinguishable from a Normal distribution. Similarly, the familiar rule that 2×2 tables should have expected counts of at least 5 for a χ^2 test comes from applying the Central Limit Theorem to binary variables.

ALTERNATIVES TO LEAST-SQUARES APPROACHES

The literature summarized above and our simulations illustrate that linear regression and the t-test can perform well with data that are far from Normal, at least in the large samples usual in public health research. In this section we examine alternatives to linear regression. In some disciplines these methods are needed to handle small samples of non-Normal data, but in reviewing their appropriateness for public health research we focus on other criteria. These methods usually come with their own sets of assumptions and they are “alternatives” to least-squares methods only when no specific summary statistic of interest can be identified, as we discuss in the next section.

We examine the Wilcoxon rank-sum test as an alternative to the t-test and the logistic and proportional odds models as alternatives to linear regression.

TABLE 1 Coverage[a] results for the mean and coefficients from multivariable regression. (Based on 1000 replicates)

% of population	N in sample	Mean	b-age	b-sex	b-EVGFP	b-IPA
1	65	88.5	89.7	96.4	88.8	93.1
2	129	90.5	89.9	96.3	88.4	91.5
5	324	92.4	89.9	97.5	91.3	93.8
7.5	487[b]	94.0	90.3	97.3	92.3	94.0
10	649[c]	94.9	91.2	97.7	92.5	94.7
15	973	95.8	92.9	98.3	94.3	96.0
20	1297	96.2	92.6	98.4	95.0	97.1

[a]Coverage is the % of time that the (nominal) 95% confidence included the true mean, out of 1000 replicates.

[b]Not always the same because some of the data are missing—468 to 500.

[c]Range from 629–669 because of missing data.

Annu. Rev. Public. Health. 2002.23:151-169. Downloaded from www.annualreviews.org
by U.S. Army Medical Research & Material Command on 01/03/14. For personal use only.

Wilcoxon and Other Nonparametric Tests

The Wilcoxon two-sample test is said to be nonparametric because no particular distribution is assumed for the data. The test simply ranks all of the data and calculates the sum of the ranks for one of the groups. It is possible to test how likely that sum would be under the null hypothesis that the two distributions were identical. The Wilcoxon test can thus be performed without distributional assumptions even in very small samples. It is sometimes described as a test for the median, but this is not correct unless the distribution in the two groups is known *a priori* to have the same shape. It is possible to construct distributions with arbitrarily different medians for which the Wilcoxon test will not detect a difference.

The Wilcoxon test is widely known to be more powerful than the t-test when the distribution of data in the two groups has long tails and has the same shape in each group but has been shifted in location. Conversely, it is less powerful than the t-test when the groups differ in the number and magnitude of extreme outlying distributions, as recognized in EPA guidelines for testing for environmental contamination in soil (33). Although its power relative to other tests depends on the details of the null and alternative hypotheses, the Wilcoxon test always has the disadvantage that it does not test for equality in any easily described summary of the data. This is illustrated by the analysis of Rascati et al. (21) in comparing overall medical costs for asthmatics prescribed steroids compared with other treatments. Although the mean cost was lower in the steroid group, a Wilcoxon test reported significantly higher costs for that group. A related disadvantage is that it is not easy to construct confidence intervals that correspond to the Wilcoxon test.

EXAMPLE As an example, we compared the outpatient cost for people who rated themselves in poor health to those in fair health ($n = 103$ and 340, respectively). The t-test showed that the mean costs in the poor and fair groups were $960 and

Annu. Rev. Public. Health. 2002.23:151-169. Downloaded from www.annualreviews.org
by U.S. Army Medical Research & Material Command on 01/03/14. For personal use only.

\$727, respectively; the mean difference is \$234; the 95% confidence interval for the difference (−\$72 to + \$540); t = 1.51; p = 0.133. The Wilcoxon test provides the information that the mean rank of costs in the poor and fair groups were 245.51 and 214.88; that the sum of ranks was 25288 versus 73058; the Wilcoxon statistic was 73058 and the p-value 0.033. The Wilcoxon test thus yielded a more significant result than the t-test, but did not provide any useful descriptive statistics. The data for the two groups did not seem to have the same shape, based on a histogram.

Logistic Regression

When the dependent variable is binary, the most common analytic method is logistic regression. In this approach the assumptions fit the data. Further, the (exponentials of the) regression parameters can be interpreted as odds ratios, which are nearly identical to relative risks when the event under study is rare.

Another possible approach is least-squares linear regression, letting *Y* be the 0/1 binary variable. Such an approach is not usually considered appropriate because *Y* is not Normally distributed; however, the Central Limit Theorem ensures that the regression coefficients will be Normally distributed for large enough samples. Regression estimates would be a weighted sum of the *Y's*, which are 0' and 1's. The usual rule for the binomial distribution is that proportions are approximately Normal if $np > 5$ and $n(1-p) > 5$, which should hold for the large data sets we are considering. Another objection to the linear regression approach is that estimated proportions can be below 0 or greater than 1. This is a problem if the goal is to predict a probability for an individual, and the sample is small. It will rarely be a problem when the goal is to assess the effects of independent variables on the outcome. A final objection is that the homoscedasticity assumption is violated, since the variance is a function of the mean. The usual rule of thumb is that if proportions are between, say, 0.2 and 0.8, the variance is approximately constant and heteroscedasticity is not a serious problem.

Linear regression might actually be preferred in some situations. Logistic regression assumes a multiplicative model, whereas linear regression provides an additive model which is sometimes more biologically plausible. The public health significance of estimates of risk and risk difference provided by a linear model is often clearer than that of odds ratios. Odds ratios can be hard to interpret when the event is not rare. This was dramatically demonstrated by a recent study of racial bias in referrals for cardiac catheterization (25), when an odds ratio of 0.6 was widely quoted in the mass media as showing a 40% percent lower chance of referral for blacks. The relative risk was actually 0.87 (26). Although logistic regression is the standard of practice for binary dependent variables, the investigator may sometimes find linear regression useful and should not be deterred by perceived problems with non-Normality.

EXAMPLE As an example, we calculated a regression to predict use of any outpatient services as a function of gender, using both logistic and OLS regression.

Annu. Rev. Public. Health. 2002.23:151-169. Downloaded from www.annualreviews.org
by U.S. Army Medical Research & Material Command on 01/03/14. For personal use only.

The linear regression of use (0/1) from sex (0/1) yielded a coefficient of 0.0663 ($t = 6.78$); the interpretation is that the use of services was 6.63 percentage points higher for women than for men. The same analysis run as a logistic regression yielded a slope of 0.402, and an estimated odds ratio of 1.495, Wald statistic $= 45.4$. The square root of the Wald statistic is 6.74, about the same as the t-statistic from linear regression.

Linear and logistic regression both give valid results in this example, but the information that the utilization among women is about 7% higher than among men may be more interpretable than the 50% relative increase in odds of utilization. It is of interest that although the odds ratio is 1.5, the relative risk is about 1.1.

Proportional Odds Model

Ordered categorical data commonly arise from assigning values to a scale that cannot be readily quantified. In our example data, participants are asked if their health is "excellent, very good, good, fair, or poor," producing a variable with five ordered categories. As there is no unique objective way to assign numerical values to these categories, it is often argued that the analysis should not depend on any particular assignment of scores.

In estimation and particularly in regression modeling, however, we would often like a single summary that describes how the outcome varies with the predictors. The behavior of a five-level category cannot be reduced to a single summary statistic without imposing some restrictions.

One popular model for analyzing ordered data is the ordinal logistic regression or proportional odds model. An ordered categorical response can be collapsed into a binary (proportional odds) model (1, 20). This model is based on the binary variables created by dichotomizing the ordinal response at any threshold C, giving the model

$$\text{logit } P[Y > C] = \alpha_C + \beta_1 X_1 + \beta_2 X_2 + \cdots + \beta_p X_p.$$

Dichotomizing at a different level C necessarily changes α_C as this is related to the proportion of outcomes above C. The proportional odds model assumes that this is the only change and that $\beta_1, \beta_2, \ldots, \beta_p$ remain the same. Although it does not make distributional assumptions, it does makes strong assumptions about the relationships between categories.

An alternative approach is to assign numerical scores to the categories, either using a default 1, 2, 3, 4, 5 or basing the scores on scientific knowledge about the underlying scale. It is then possible to analyze ordered categorical data by ordinary least squares regression. If two groups have the same probability distribution, the mean measurement will be the same, no matter how numbers are assigned to each possible level of outcome. Furthermore, if there is in fact a tendency for higher measurements to predominate in one group more than the other, the mean will tend to be shifted in that direction. In this setting, however, there is no clear scientific interpretation of the size of a nonzero difference in the means, leading

Annu. Rev. Public. Health. 2002.23:151-169. Downloaded from www.annualreviews.org
by U.S. Army Medical Research & Material Command on 01/03/14. For personal use only.

to the difficulties in interpretation similar to those with the Wilcoxon and other nonparametric tests. There is also a potential problem with heteroscedasticity in assigning scores, but as with binary data, this is only likely to be important if a large majority of the observations are in the highest or lowest categories.

EXAMPLE We predicted whether an adult's health would be excellent, very good, good, fair, or poor based on whether or not he or she had less than a high school education. We examined the assumption that dichotomization at any level of EVGFP gave the same level. If we compared excellent to the other four categories, the odds ratio (OR) was 0.50; breaking at very good, the OR was 0.45; at good, the OR was 0.42; and the OR from dichotomizing below Fair was 0.70. The last odds ratio was not significantly different from the others (95% confidence interval of 0.38 to 1.30). The ordered logistic regression gave a common odds ratio of 0.46. The interpretation is that wherever one cuts the health variable, the odds of being in the healthy group are about half as high for persons with only a high school education. As above, the odds ratio is not the same as the relative risk, since being in the "high" health category is not a rare outcome.

A linear regression where Y takes on the values from 1 (poor) to 5 (excellent) achieves a coefficient of -0.42 and a t-statistic of -7.55. The interpretation is that adults with low education are about a half-category lower in health than those with more education. The t-statistics for the linear and the ordered logistic regressions are nearly identical. While neither model is ideal in terms of scientific interpretation, it is easier to give a precise description of the results for the linear model than for the proportional odds model. In the absence of any strong reason to trust the proportional odds assumption the linear model would be a sensible default analysis.

REASONS FOR CHOOSING AN ESTIMATOR OR TEST

It is rarely necessary to worry about non-Normality of outcome variables. It is necessary to worry about heteroscedasticity in linear regression, though, as the example shows, even with significant heteroscedasticity the performance of linear regression is often good.

The fact that these methods are often valid does not mean that they are ideal in all cases, merely that the reasons for choosing other analyses are different from those often given. The t-test and linear regression both estimate differences in the mean of the outcome. In some cases, this is precisely what is of interest: Health care suppliers, for example, care about the mean cost of care. In other cases, some other summary is appropriate: Median income or percentage living in poverty may be more relevant in considering access to health care. Although costs and income are both measured in dollars, and both have strongly skewed distributions, different questions lead to different choices of summary statistic.

In other examples, the choice between mean, median, and percentage below some threshold may be less obvious. The decision of whether to base a data analysis

Annu. Rev. Public. Health. 2002.23:151-169. Downloaded from www.annualreviews.org
by U.S. Army Medical Research & Material Command on 01/03/14. For personal use only.

on a particular summary measure, if any, should be based on the following criteria (in order of importance): clinical or scientific relevance of the summary measure, scientific plausibility that the groups would differ with respect to the summary measure, and statistical precision with which the groups can be compared using the summary measure.

If the question under investigation identifies the preferred analysis, as with comparing total medical care costs, other criteria are irrelevant. It may be easier to estimate differences in, say, the median, but differences in the median need not be a good guide to differences in the mean and so are of little interest. This is what happened in the analysis of Rascati et al. (21) discussed above, where a Wilcoxon test indicated significant cost differences in the opposite direction to the difference in mean costs.

On the other hand, we may not know which summary measure is most important, but have some idea which summary measure is most likely to be affected. Consider a cholesterol-lowering drug, where it might be the case that the treatment is thought to work only in individuals whose initial cholesterol measurement is extremely high. In this setting, there may be no difference between treated and untreated populations with respect to the median cholesterol level, though the mean cholesterol level in the treated group would be lower, as would the proportion of individuals exceeding some high threshold. However, if in that same example the drug is thought not to have effect in individuals with the most severe disease, then neither the median cholesterol level nor the proportion of individuals having cholesterol higher than, say, 350 mg/dl might differ between the control and treatment groups. The mean cholesterol level might still differ between the groups owing to the segment of the population with moderately high cholesterol for whom the treatment was effective. A t-test would then detect this difference, but a comparison of medians might not.

Finally, we may have no real knowledge of which summary statistic is most likely to differ between different groups of people. In this case, we may still have a preference based on statistical sensitivity or on convenience or other factors. For example, if a measurement (such as serum cholesterol) has a very long right tail, the mean is hard to estimate reliably. This would be a valid argument against using a t-test if we had no particular interest in the mean as a summary and no particular knowledge of how cholesterol varies between groups of people. The median or the geometric mean might be better summaries, leading to a different test or to a t-test based on transformed data.

This discussion has been phrased in terms of the t-test, but the same criteria apply in considering alternatives to linear regression. There are many alternative regression methods, like the proportional odds model for categorical data or more robust median regressions for long-tailed data. These quantify the effects of a predictor variable in different ways. Sometimes it will be possible to identify the desired method based on the scientific question to be answered. On other occasions we may know whether the effect is likely to be a small increase in most values (perhaps favoring a robust regression) or a large increase in a few outliers (which would be ignored by a robust regression).

SUMMARY AND CONCLUSIONS

The t-test and least-squares linear regression do not require any assumption of Normal distribution in sufficiently large samples. Previous simulations studies show that "sufficiently large" is often under 100, and even for our extremely non-Normal medical cost data it is less than 500. This means that in public health research, where samples are often substantially larger than this, the t-test and the linear model are useful default tools for analyzing differences and trends in many types of data, not just those with Normal distributions. Formal statistical tests for Normality are especially undesirable as they will have low power in the small samples where the distribution matters and high power only in large samples where the distribution is unimportant.

While the large-sample properties of linear regression are well understood, there has been little research into the sample sizes needed for the Normality assumption to be unimportant. In particular, it is not clear how the necessary sample size depends on the number of predictors in the model.

The focus on Normal distributions can distract from the real assumptions of these methods. Linear regression does assume that the variance of the outcome variable is approximately constant, but the primary restriction on both methods is that they assume that it is sufficient to examine changes in the mean of the outcome variable. If some other summary of the distribution is of greater interest, then the t-test and linear regression may not be appropriate.

APPENDIX 1

The Central Limit Theorem

The classical version of the Central Limit Theorem taught in introductory statistics courses deals with averages of identically distributed data. This suffices for the t-test but not for linear regression, where the regression coefficients are computed from averages of the outcome multiplied by the covariates. To cover both cases we use the Lindeberg-Feller Central Limit Theorem (6). An approximate translation of this result is that if $Y_1, Y_2, \ldots Y_n$ are a large collection of independent random variables with variances $s_1^2, s_2^2, \ldots, s_n^2$ the average

$$\overline{Y} = \frac{1}{n}\sum_{i=1}^{n} Y_i$$

is approximately Normally distributed, with mean equal to the average of the means of the *Y*s and variance equal to the average of their variances, under two conditions:

1. The variance of any single observation is small compared to the sum of the variances.
2. The number of outcomes that are extreme outliers, more than $\sqrt{n}$ standard deviations away from their mean, is small.

Annu. Rev. Public. Health. 2002.23:151-169. Downloaded from www.annualreviews.org
by U.S. Army Medical Research & Material Command on 01/03/14. For personal use only.

These conditions both restrict the impact any single observation can have on the average. Extreme outliers and very unequal variances (such as might be caused by outlying covariate values in linear regression) are allowed, but imply that larger sample sizes are needed.

This result does not answer the perennial question "How large is large?," and theoretical results are not particularly helpful. In order to understand how the required sample size varies for different sorts of data, we need to rely on simulations that reflect the sort of data we typically use. We do know that the important features of such a simulation are how the sample size relates to the differences in variance and the prevalence of extreme outliers; this information will help us generalize from the simulations to other sorts of data.

Annu. Rev. Public. Health. 2002.23:151-169. Downloaded from www.annualreviews.org
by U.S. Army Medical Research & Material Command on 01/03/14. For personal use only.

The t-Statistic

Let $\overline{Y}_1$ and $\overline{Y}_2$ be the mean of Y in groups 1 and 2 respectively. By the Central Limit Theorem, if n_1 and n_2 are large enough $\overline{Y}_1 \sim N(\mu_1, \sigma_1^2)$ and $\overline{Y}_2 \sim N(\mu_2, \sigma_2^2/n_2)$, so

$$\overline{Y}_1 - \overline{Y}_2 \sim N\left(\mu_1 - \mu_2, \frac{\sigma_1^2}{n_1} + \frac{\sigma_2^2}{n_2}\right)$$

and thus

$$\frac{\overline{Y}_1 - \overline{Y}_2}{\sqrt{\frac{\sigma_1^2}{n_1} + \frac{\sigma_2^2}{n_2}}} \sim N(\mu_1 - \mu_2, 1).$$

Now in a large sample, $s_1^2$and s_2^2 are close to σ_1^2 and σ_2^2, so we may replace the population variance by the sample variance to arrive at the unequal-variance form of the t-statistic.

Linear Regression

The parameter estimates in least-squares linear regression are given by the matrix formula

$$\hat{\beta} = (X^T X)^{-1} X^T Y$$

This formula shows that each coefficient is a weighted average of the Y values with weights that depend in a complicated way on the covariates X. That is, we can write each coefficient as

$$\hat{\beta}_j = \frac{1}{n} \sum_{i=1}^{n} w_i Y_i.$$

This is an average of variables $w_i Y_i$ that have different distributions depending on X, but the Central Limit Theorem still applies. In this case extreme values of Y or of X will increase the required sample size.

Visit the Annual Reviews home page at www.annualreviews.org

LITERATURE CITED

1. Agresti A. 1990. *Categorical Data Analysis*. New York: Wiley
2. Altman DG. 1991. *Practical Statistics for Medical Research*. London: Chapman & Hall
3. Barber JA, Thompson SG. 2000. Analysis of cost data in randomized trials: an application of the non-parametric bootstrap. *Statist. Med.* 19:3219–36
4. Barrett JP, Goldsmith L. 1976. When is n sufficiently large? *Am. Stat.* 30:67–70
5. Berk RA. 1990. A primer on robust regression. In *Modern Methods of Data Analysis*, ed. J Fox, JS Long, pp. 292–324. Newbury Park, CA: Sage
6. Billingsley P. 1995. *Probability and Measure*. New York: Wiley. 3rd. ed.
7. Chen L. 1995. Testing the mean of skewed distributions. *J. Am. Stat. Assoc.* 90:767–72
8. Cohen ME, Arthur JS. 1991. Randomization analysis of dental data characterized by skew and variance heterogeneity. *Comm. Dent. Oral.* 19:185–89
9. Davison AC, Hinkley DV. 1997. *Bootstrap Methods and their Application*. Cambridge: Cambridge Univ. Press
10. Diehr P, Madden C, Martin DP, Patrick DL, Mayers M. 1993. Who enrolled in a state program for the uninsured: Was there adverse selection? *Med. Care* 31:1093–105
11. Diehr P, Yanez D, Ash A, Hornbrook M, Lin DY. 1999. Methods for analyzing health care utilization and costs. *Annu. Rev. Public Health* 20:125–44
12. Efron B, Tibshirani R. 1993. *An Introduction to the Bootstrap*. New York: Chapman & Hall
13. Fisher LD, van Belle G. 1993. *Biostatistics: A Methodology for the Health Sciences*. New York: Wiley
14. Gayen AK. 1949. The distribution of Student's t in random samples of any size drawn from non-normal universes. *Biometrika* 36:353–69
15. Geary RC. 1936. The distribution of Student's ratio for non-normal samples. *J. R. Stat. Soc.* 3 (Suppl.):178–84
16. Goodall C. 1983. M-estimators of location: an outline of the theory. In *Understanding Robust and Exploratory Data Analysis*, ed. DC Hoaglin, F Mosteller, JW Tukey, pp. 339–41. New York: Wiley
17. Insightful. 2000. *S-PLUS 2000.* Seattle, WA: Insightful Corp.
18. Kleinbaum DG, Kupper LL, Muller KE, Nizam A. 1998. *Applied Regression Analysis and Multivariable Methods*. Pacific Grove, CA: Duxbury. 3rd ed.
19. Martin D, Diehr P, Cheadle A, Madden C, Patrick D, Skillman S. 1997. Health care utilization for the "newly insured": results from the Washington Basic Health Plan. *Inquiry* 34:129–42
20. McCullagh P. 1980. Regression models for ordinal data. *J. R. Stat. Soc. B* 42:109–42
21. Rascati KL, Smith MJ, Neilands T. 2001. Dealing with skewed data: an example using asthma-related costs of Medicaid clients. *Clin. Ther.* 23:481–98
22. Ratcliffe JF. 1968. The effect on the t distribution of non-normality in the sampled population. *Appl. Stat.* 17:42–48
23. Sawilowsky SS, Blair RC. 1992. A more realistic look at the robustness and type II error properties of the t test to departures from population normality. *Psychol. Bull.* 111:352–60
24. Sawilowsky SS, Hillman SB. 1993. Power of the independent samples t test under a prevalent psychometric measure distribution. *J. Consult. Clin. Psychol.* 60:240–43
25. Schulman KA, Berlin JA, Harless W, Kerner JF, Sistrunk S, et al. 1999. The effect of race and sex on physicians' recommendations for cardiac catheterization. *New Engl. J. Med.* 340:618–26
26. Schwartz LM, Woloshin S, Welch HG.

Annu. Rev. Public. Health. 2002.23:151-169. Downloaded from www.annualreviews.org by U.S. Army Medical Research & Material Command on 01/03/14. For personal use only.

1999. Misunderstandings about the effect of race and sex on physicians' referrals for cardiac catheterization. *New Engl. J. Med* 341:289–93

27. Skovlund D, Fenstad GU. 2001. Should we always choose a nonparametric test when comparing two apparently nonnormal distributions? *J. Clin. Epidemiol.* 54:86–92
28. Smith DE. 1959. *A Source Book in Mathematics*. Dover
29. StataCorp. 2001. *Stata Statistical Software: Release 7.0*. College Station, TX: Stata Corp.
30. Stonehouse JM, Forrester GJ. 1998. Robustness of the t and U tests under combined assumption violations. *J. Appl. Stat.* 25:63–74
31. Stuart A, Ord K, Arnold S. 1999. *Kendall's Advanced Theory of Statistics: 2A Classical Inference and the Linear Model*. London: Arnold. 6th ed.
32. Sullivan LM, D'Agostino RB. 1992. Robustness of the t test applied to data distorted from normality by floor effects. *J. Dent. Res.* 71:1938–43
33. US EPA. 1994. *Statistical Methods for Evaluating the Attainment of Cleanup Standards, Vol. 3: Reference-based Standards for Soils and Solid Media*. EPA/230-R-94-004. Off. Policy Plan. Eval. US EPA, Washington, DC
34. Zimmerman DW. 1998. Invalidation of parametric and nonparametric statistical tests by concurrent violation of two assumptions. *J. Exp. Educ.* 67:55–68
35. Zimmerman DW, Zumbo DW. 1992. Parametric alternatives to the student t test under violation of normality and homogeneity of variance. *Percept. Motor. Skill.* 74:835–44

Annu. Rev. Public. Health. 2002.23:151-169. Downloaded from www.annualreviews.org
by U.S. Army Medical Research & Material Command on 01/03/14. For personal use only.

Annual Review of Public Health
Volume 23, 2002

CONTENTS

EPIDEMIOLOGY AND BIOSTATISTICS

HALYs and QALYs and DALYs, Oh My: Similarities and Differences in Summary Measures of Population Health, *Marthe R. Gold, David Stevenson, and Dennis G. Fryback* 115

The Importance of the Normality Assumption in Large Public Health Data Sets, *Thomas Lumley, Paula Diehr, Scott Emerson, and Lu Chen* 151

Gambling and Related Mental Disorders: A Public Health Analysis, *Howard J. Shaffer and David A. Korn* 171

Thinking Outside the Box: Recent Advances in the Analysis and Presentation of Uncertainty in Cost-Effectiveness Studies, *Andrew H. Briggs, Bernie J. O'Brien, and Gordon Blackhouse* 377

ENVIRONMENTAL AND OCCUPATIONAL HEALTH

Socioeconomic Status and Health: The Potential Role of Environmental Risk Exposure, *Gary W. Evans and Elyse Kantrowitz* 303

Effects of Smoking Restrictions in the Workplace, *Ross C. Brownson, David P. Hopkins, and Melanie A. Wakefield* 333

The Future of Benefit-Cost Analyses of the Clean Air Act, *Alan Krupnick and Richard Morgenstern* 427

PUBLIC HEALTH PRACTICE

Public Health Quality Measurement: Concepts and Challenges, *Stephen F. Derose, Mark A. Schuster, Jonathan E. Fielding, and Steven M. Asch* 1

The Effectiveness of State-Level Tobacco Control Interventions: A Review of Program Implementation and Behavioral Outcomes, *Michael Siegel* 45

Challenges in Motor Vehicle Safety, *Patricia F. Waller* 93

Effects of Smoking Restrictions in the Workplace, *Ross C. Brownson, David P. Hopkins, and Melanie A. Wakefield* 333

Tuberculosis, *Parvathi Tiruviluamala and Lee B. Reichman* 403

Annu. Rev. Public. Health. 2002.23:151-169. Downloaded from www.annualreviews.org
by U.S. Army Medical Research & Material Command on 01/03/14. For personal use only.

Social Environment and Behavior

Direct Marketing of Pharmaceuticals to Consumers, *Alan Lyles* 73

The Public Health Impact of Alzheimer's Disease, 2000–2050: Potential Implication of Treatment Advances, *Philip D. Sloane, Sheryl Zimmerman, Chirayath Suchindran, Peter Reed, Lily Wang, Malaz Boustani, and S. Sudha* 213

Dietary Interventions to Prevent Disease, *Deborah J. Bowen and Shirley A. A. Beresford* 255

The Macroeconomic Determinants of Health, *S. V. Subramanian, Paolo Belli, and Ichiro Kawachi* 287

Socioeconomic Inequalities in Injury: Critical Issues in Design and Analysis, *Catherine Cubbin and Gordon S. Smith* 349

Health Services

Cascade Effects of Medical Technology, *Richard A. Deyo* 23

Direct Marketing of Pharmaceuticals to Consumers, *Alan Lyles* 73

Morbidity and Mortality from Medical Errors: An Increasingly Serious Public Health Problem, *David P. Phillips and Charlene C. Bredder* 135

Utilization Management: Issues, Effects, and Future Prospects, *Thomas M. Wickizer and Daniel Lessler* 233

Indexes

Subject Index 449

Cumulative Index of Contributing Authors, Volumes 14–23 473

Cumulative Index of Chapter Titles, Volumes 14–23 478

Errata

An online log of corrections to *Annual Review of Public Health* chapters (if any have yet been occasioned, 1997 to the present) may be found at http://publhealth.annualreviews.org/

Annu. Rev. Public. Health. 2002.23:151-169. Downloaded from www.annualreviews.org by U.S. Army Medical Research & Material Command on 01/03/14. For personal use only.
