# Supplementary material for: Combat-related bridge synostosis versus traditional transtibial amputation: comparison of military-specific outcomes
Source: Strategies Trauma Limb Reconstr. 2015 Dec 7;11(1):5–11. doi: 10.1007/s11751-015-0240-4 (PMC4814387; doi:10.1007/s11751-015-0240-4)
Supplement: Supplementary file 2 — Supplementary material 2 (RTF 6 kb) [file 11751_2015_240_MOESM2_ESM.rtf]

Contingency Analysis of trt By rank
Weight: count


Contingency Table
rank By trt
Count
Total %
Col %
Row %	b	e		
j	128
26.78
31.53
90.78	13
2.72
18.06
9.22	141
29.50	
n	248
51.88
61.08
82.94	51
10.67
70.83
17.06	299
62.55	
o	30
6.28
7.39
78.95	8
1.67
11.11
21.05	38
7.95	
	406
84.94	72
15.06	478	

Tests
N	DF	 -LogLike	RSquare (U)	
478	2	3.0676452	0.0151	

Test	ChiSquare	Prob>ChiSq	
Likelihood Ratio	6.135	0.0465*	
Pearson	5.757	0.0562	

Cochran Armitage Trend Test
Asymptotic Test
Z	Prob<Z	Prob>|Z|	
 -2.34754	0.0094*	0.0189*	
